# Adaptation of a microbead assay for the easy evaluation of traditional anti-sickling medicines: application to DREPANOSTAT and FACA

**DOI:** 10.1080/13880209.2018.1501585

**Published:** 2018-09-28

**Authors:** Joran Villaret, Guillaume Marti, Frédérique Dubois, Karine Reybier, Noémie Gaudre, Mohamed Haddad, Alexis Valentin

**Affiliations:** a Pharma-Dev, UMR 152, Université de Toulouse, IRD, UPS, Toulouse, France;; b Service Hématologie, Pôle Biologie, Institut Universitaire du Cancer de Toulouse – Oncopole, Toulouse, France;; c Service Médecine Vasculaire, Pôle Cardiovasculaire et Métabolique, Hôpital Rangueil, Toulouse, France

**Keywords:** Sickle cell anemia, metabolomics, UHPLC-HRMS, traditional medicines, *Zanthoxylum zanthoxiloides*, *Calotropis procera*

## Abstract

**Context:** Sickle cell disease is a common inherited blood disorder affecting millions of people worldwide. Due to lack of progress in drug discovery for a suitable treatment, sufferers often turn to traditional medicines that take advantage of the plant extracts activity used by traditional healers.

**Objective:** This study optimizes an anti-sickling screening test to identify preparations capable of reverting sickle cells back to the morphology of normal red blood cells. We focused on the miniaturization and practicability of the assay, so that it can be adapted to the laboratory conditions commonly found in less developed countries.

**Materials and methods:** We tested two traditional anti-sickling herbal medicines, FACA^®^ and DREPANOSTAT^®^, composed of *Zanthoxylum zanthoxyloides* (Lam.) Zepern. & Timler (Rutaceae) and *Calotropis procera* (Aiton) Dryand. (Apocynaceae) at screening concentrations of hydroethanol extracts from 0.2 to 1 mg/mL. Potential bioactive molecules present in the extracts were profiled using Ultra High Performance Liquid Chromatography coupled with High Resolution Mass Spectrometry (UHPLC-HRMS/MS) method, identified through HRMS, MS/MS spectra and *in silico* fragmentation tools.

**Results:** Hydroethanol extracts of FACA^®^ and DREPANOSTAT^®^ showed low anti-sickling activity, inhibiting less than 10% of the sickling process. The UHPLC-HRMS/MS profiles identified 28 compounds (18 in FACA^®^ and 15 in DREPANOSTAT^®^, including common compounds) among which l-phenylalanine is already described as potential anti-sickling agent. When used as positive control, 7 mg/mL phenylalanine reduced the sickled RBC to 52%.

**Discussion and conclusions:** This assay has been optimized for the easy screening of plant extracts or extracted compounds from bioassay guided fractionation, valuable to laboratories from less developed countries.

## Introduction

Hemoglobin is the main component of red blood cells (RBC), and is responsible for transporting oxygen throughout the body. Hemoglobin A (Hb A), the most common form, is composed of two *α*- and two *β*-globin chains. Normal RBCs are mainly composed of Hb A, and have a biconcave shape which allows them to pass through small blood vessels. Sickle cell anemia is a genetic disorder of hemoglobin. The mutation triggers a single amino acid substitution of glutamic acid for valine at the sixth position on the *β*-globin chain to generate hemoglobin S (Hb S), which polymerizes in hypoxic conditions to give rise to abnormal sickle shaped RBC (Pauline et al. 2013). The disease is inherited in an autosomal recessive way. Homozygous patients are often affected by hypoxia, vaso-occlusive pain crises and strokes because of the aggregation of sickled RBC in the microcirculation (Nurain et al. 2017). Approximately 300,000 newborns are affected each year, and 5% of the global population is healthy carriers; this can reach up to 25% in some African areas exposed to malaria (WHO 2011). Moreover, the total number of patients affected by sickle cell disease is expected to increase due to the remarkable level of protection that the sickle cell provides against malaria (Piel et al. 2017). Indeed, people affected by sickle cell anemia in malaria endemic areas seem to have a significantly increased lifespan, which leads to positive natural selection. Due to slave trading and migration, sickle cell disease has spread to most developed countries, and in these malaria-free countries the life expectancy for patients with sickle cell disease is reduced by approximately 30 years (Piel et al. 2017). As it is a genetic disease, there is no pharmacological cure for sickle cell anemia; however various techniques are currently used to treat the symptoms, including blood transfusion and hydroxyurea. This chemotherapeutic drug is known to decrease the occurrence of vaso-occlusive crises notably by stimulating the production of fetal hemoglobin (Hb F) in RBC, however the detailed biological mechanism has not yet been elucidated (Brandow and Panepinto 2010). Treatment with hydroxyurea also has several disadvantages including risks factors associated with its long term usage, moreover it is an expensive treatment especially for patients from less developed countries. Hence, African people are mainly using traditional medicine to treat sickle cell anemia, this led to the production of NIPRISAN^®^ (a combination of four plants) in Nigeria (Wambebe et al. 2001), DREPANOSTAT^®^ in Togo and FACA^®^ in Burkina Faso. Various investigations have been carried out previously (Queiroz et al. 2006) to evaluate and isolate potential anti-sickling compounds present in these plants (Abu et al. 1981). Moreover, a new molecule called burkinabin has been isolated and identified from plants used in traditional medicines in sub-Saharan countries (Ouattara et al. 2004, 2009).

The objective of our study was first to develop a simple screening test easily transposable to sub-Saharan countries, to evaluate the potential anti-sickling activity of traditional plant extracts. The tests were performed with aqueous ethanol extracts of FACA^®^ and DREPANOSTAT^®^ composed of *Zanthoxylum zanthoxyloides* (Lam.) Zepern. & Timler (Rutaceae) and *Calotropis procera* (Aiton) Dryand. (Apocynaceae). Then, we aimed to decipher the complex molecular blends of these complementary alternative medicines (CAMs) using UHPLC-HRMS/MS fingerprints, and tried to identify components by comparing the experimental mass spectrometry fragmentation patterns to an *in silico* fragmentation.

## Materials and methods

### Plant materials

*Zanthoxylum zanthoxyloides* and *C. procera* were obtained from FACA^®^ and DREPANOSTAT^®^. FACA^®^ is composed of a mixture of these two plants (Ouedraogo et al. 2011), whereas DREPANOSTAT^®^ is composed only of *Z. zanthoxyloides* (Pousset 2006). Plants were washed, dried, powdered and then encapsulated to obtain these two drugs, usually administered orally (Matu 2011).

### Extraction

The contents of 400 capsules of FACA^®^ and 90 capsules of DREPANOSTAT^®^ were brought together to give respectively 61.00 g and 22.99 g of powder. Vegetal powders were then macerated in 320 mL of water/ethanol (50/50) (Fisher Scientific, France). The extracts obtained were evaporated under reduced pressure in order to obtain a small volume of suspension in water. Aqueous solutions were finally frozen and lyophilized to obtain aqueous ethanol extract powders (7.72 g for FACA^®^ and 3.60 g for DREPANOSTAT^®^).

### Blood samples

Blood samples were obtained from adult homozygous patients affected by sickle cell disease at the hospital of Toulouse (Purpan, France). For each patient, 5 mL of blood was collected and stored at 4 °C in ethylaminetetraacetic acid (EDTA) tubes. Heterozygous patients and patients who recently benefited from a blood transfusion were excluded from the experiment. Our study was approved by the local Ethics Committee, oral and written informed consent was obtained from the patients before blood collection. Blood samples (5 mL from each volunteer) were also collected from healthy volunteers to serve as positive controls.

### Preparation of the assay

Blood samples were centrifuged at 550 *g* for 10 min, in order to separate and remove plasma and buffy coat from the RBC. The RBC were then washed twice in Roswell Park Memorial Institute Medium 1640 (RPMI) (Fisher Scientific, France) and stored at 4 °C for a maximum period of one month. Sephacryl S-500 HR microbeads (Sigma-Aldrich, France) were stored at 4 °C in ethanol suspension and were washed twice in phosphate-buffered saline (PBS) before experiments. Finally, the microbeads were suspended in PBS to obtain a 50/50 volume of suspension.

### Principle of the microbead assay

Our assay was adapted from the high throughput screening test developed by Pais et al. (2009) in order to lower the reliance upon robotic equipment which are not readily available in less developed countries, and also to reduce the overall cost of the assay. The principle of the test consists of evaluating the capacity of the RBC to pass through packed Sephacryl S-500 HR chromatography beads upon centrifugation: essentially normal RBC can pass through, whereas sickled RBC are blocked at the top of the packed beads. Specifically, 250 µL of microbead suspension was introduced into a transparent polypropylene flat bottom 96-well plate (Thermofisher Scientific, France). The plate was centrifuged for 5 min at 170 *g* to pack the beads at the bottom of each well. Then, 20 µL of normal RBC or sickled RBC were then added to the surface of the packed microbeads. The plate was placed on a plate shaker (TITRAMAX 100, Heidolph Instruments, France) at 170 *g* for 30 min, and then the plate was once more centrifuged at 170 *g* for 5 min. The plate was placed again on the plate shaker for 15 min. The percentage of sickled RBC was evaluated by measuring the color at the bottom of the well.

### Data analysis

**Figure 1.**
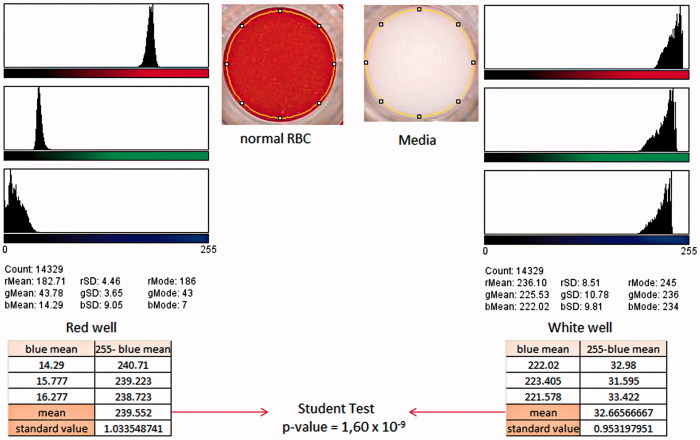
Analysis of the scanned image of the bottom of a 96-well plate by ImageJ^®^, the red well was obtained with normal RBC following the protocol described in the text. The white well was obtained using the same protocol with RBC-free media. The abbreviations r, g and b were used for the colors red, green and blue, respectively.

Results were obtained using a scanned image of the bottom of the 96-well plate (CanoScan 4400 F, Canon France) at a resolution of 600 dots per inch (DPI), to monitor the color of each well. We used ImageJ^®^ software to record a 14,329-pixel circle which made it possible to quantify for each pixel of the selected area, the intensity of red, green and blue colors, and automatically give means and standard deviation values for each color (Schindelin et al. 2015). For the purpose of our study we used the mean intensities for blue, as the differences between the positive (red well) and negative (white well) controls were the greatest for this color (see Figure 1). After that, the mean intensities obtained for blue were subtracted from 255, which is the maximum value measured by this software. This subtraction allowed us to obtain a value reflecting the quantity of RBC present at the bottom of the well. The experiments were performed in triplicate and the new mean values obtained were compared using Student’s *t*-test.

### Induction of sickling

As sickling occurs only during hypoxic conditions, sodium metabisulfite was used to induce the morphological modification of RBC. Its strong reducing effect prevents the fixation of oxygen on hemoglobin, thus mimicking anaerobic conditions. A freshly prepared solution of 2% sodium metabisulfite (Sigma-Aldrich, France) in PBS was used to obtain the desired volume of solution containing Hb S RBC in an Eppendorf tube incubated at 37 °C (Mpiana et al. 2010).

### Hemolytic assay

We incubated 200 µL of 2.5% erythrocytes in suspension in PBS with 200 µL of each of the plant extracts in suspension in PBS (2 mg/mL) at 37 °C for 30 min, to give a final concentration of the plant extracts of 1 mg/mL. After centrifugation (380 *g*, 5 min), the hemoglobin content in the supernatant (triplicate of 75 µL) was measured at 450 nm using a multiwell spectrophotometer (Eon, Biotek, France). The absorbance of the plant extracts and RBC suspended in PBS were measured as controls. The results were then compared to a positive control sample containing erythrocytes in distilled water (Haddad et al. 2004).

### LC-(ESI)-HRMS/MS analysis

We solubilized 75 mg of plant extract using an ultrasonic bath (FisherBrand, France) in an 80/20 ethanol/water solution. The solutions were then centrifuged to collect the supernatant which contained soluble metabolites. All extracts were profiled using a UHPLC-LTQ-Orbitrap XL instrument (Ultimate 3000, Thermo Fisher Scientific, Hemel Hempstead, UK). Mass detection was performed using an electrospray source in positive and negative ionization (ESI) modes at 15,000 resolving power. The mass scanning range was *m/z* 100-1500 Da. The capillary temperature was 300 °C and ISpray voltage was fixed at 4.2 (positive mode) and 3.0 kV (negative mode). Mass measurement was externally calibrated before starting the experiment. Each full MS scan was followed by data dependent MS/MS on the four most intense peaks using stepped collision-induced dissociation (35% normalized collision energy, isolation width 2 Da). The LC-MS system was run in binary gradient mode using a BEH C18 ACQUITY column (1.7 µM, 2.1 mm ×150 mm, Waters, MA, USA) equipped with a guard column. Mobile phase A (A) was 0.1% formic acid (FA) in water and mobile phase B (B) was 0.1% FA in acetonitrile (Fisher Scientific, France). Gradient conditions were: 0–0.5 min 95% phase A and 5% phase B; 0.5–22 min 50% A and 50% B; 22–32 min 5% A and 95% B; 32–35 min 5% A and 95% B and 35–40 min was used to return to initial column conditions for further analyses. The flow rate was 0.3 mL/min, column temperature 40 °C and the injection volume was 2 µL.

### In silico fragmentation analysis

First, the UHPLC-LTQ-Orbitrap raw profiles of the extracts in negative and positive modes were processed using MS-DIAL version 2.56 (Tsugawa et al. 2015). Mass signal was extracted between 100 and 1500 Da from 2 to 37 min. The respective MS1 and MS2 tolerance was set to 0.001 and 0.05 Da, in centroid mode. The detection threshold was optimized for peak detection and finally set at 500,000. Adducts and complexes were identified in order to exclude them from the final peak list. Peaks detected using a blank sample were also deleted from the final peak list to prevent false positives. Then, the molecular formulas of significant features were calculated using MS-FINDER version 2.10 (Tsugawa et al. 2016). We used various parameters to reduce the number of potential candidates such as element selection exclusively including C, H, O and N; the mass tolerance fixed to 10 ppm and the isotopic ratio tolerance set to 20%. Furthermore, we used *in silico* MS/MS fragmentation for compound annotation. Firstly, in-house databases were interrogated based on genera *Zanthoxylum* and *Calotropis*, then family databases were screened (Rutaceae and Apocynaceae, respectively). Finally, natural product databases focused on plants were selected: Universal Natural Products Database (UNPD), KNApSAck, PlantCyc, Dictionary of Natural Products (DNP, CRC press, v25:2) and CheBI. The results were presented as a list of compounds sorted according to the closeness of the match materialized by a score value which encompassed uncertainty on accurate mass, the isotopic pattern score and the experimental MS/MS fragmentation mirrored to *in-silico* matches. Only structures with a total score above 7.5 and with a structure in agreement between databases were retained (Table 1).

**Table 1.** Formula, names and PubChem ID of identified metabolites as classified by the MS-FINDER software.

ID	Retention time	Exact Mass	Ionisation mode	Error (mDa)	Formula finder	name	scoring	Pubchem ID
**DREPANOSTAT**								
1	11.55	491.1192	[M−H]−	0.2998	C23H24O12	Burkinabin B	7.96	23250813
2	12.19	491.1186	[M−H]−	0.8998	C23H24O12	Burkinabin A/C	7.4243	23250812/23250814
3	9.93	367.1031	[M−H]−	0.3558	C17H20O9	Phellodenol H	8.0524	16088228
4	34.34	471.3471	[M−H]−	0.8836	C30H48O4	Bourjotinolone A	7.5365	91895451
5	6.35	325.0922	[M−H]−	0.6911	C15H18O8	1-O-(4-Coumaroyl)-beta-D-glucose	7.6869	14158117
6	11.59	609.1813	[M + H]+	0.0968	C28H32O15	Diosmin	7.9633	5281613
7	14.45	593.1865	[M + H]+	−0.0178	C28H32O14	Diosmin	9.0426	5281613
8	3.07	166.0863	[M + H]+	−0.0450	C9H11NO2	l-Phenylalanine	8.9394	6140
9	6.12	355.1026	[M + H]+	−0.2414	C16H18O9	Undulatoside A	8.1954	5321494
10	19.26	256.1332	[M + H]+	0.0052	C16H17NO2	Dihydroalatamide	8.7494	3083797
11	10	465.1027	[M + H]+	0.0525	C21H20O12	Quercimeritrin	8.3672	5282160
12	5.02	205.0972	[M + H]+	−0.0459	C11H12N2O2	l-Tryptophan	8.3011	6305
13	24.56	271.0963	[M + H]+	0.1854	C16H14O4	Imperatorin	7.6442	10212
14	27.95	455.3524	[M + H]+	−0.4282	C30H46O3	Katononic Acid	8.1143	9981416
15	22.2	244.1333	[M + H]+	−0.0948	C15H17NO2	Atanine	7.9502	10014667
16	25.72	408.1441	[M + H]+	0.0638	C23H21NO6	8-Carboxymethyldihydrochelerythrine	7.9495	N/A
17	10.96	260.1282	[M + H]+	−0.0801	C15H17NO3	Geibalansine	7.7702	11086467
2	12.2	493.1348	[M + H]+	−0.7473	C23H24O12	Burkinabin A/C	7.7514	23250812/23250814
18	10.63	197.1174	[M + H]+	−0.1792	C11H16O3	Loliolide	7.5468	100332
**FACA**								
1	11.54	491.1187	[M−H]−	0.7998	C23H24O12	Burkinabin B	7.7473	23250813
3	8.41	367.1031	[M−H]−	0.3558	C17H20O9	Phellodenol H	7.7423	16088228
19	8.9	653.1716	[M−H]−	0.7232	C29H34O17	Rhamnazin 3-sophoroside	7.7352	N/A
8	3.18	166.0862	[M + H]+	0.0550	C9H11NO2	l-Phenylalanine	8.9743	6140
20	10.8	523.1445	[M + H]+	0.1173	C24H26O13	3,7,4'-Trimethylquercetin 2'-*O*-*β*-d-glucoside	8.0079	N/A
21	17.27	316.1544	[M + H]+	−0.0654	C18H21NO4	3-Hydroxy-*N*-methylcoclaurine	7.943	440591
16	25.72	408.1439	[M + H]+	0.2638	C23H21NO6	8-Carboxymethyldihydrochelerythrine	7.9427	N/A
22	9.71	549.2692	[M + H]+	0.2239	C29H40O10	Calotoxin	7.7212	56840852
23	2.1	132.1017	[M + H]+	0.2051	C6H13NO2	l-Isoleucine	7.8287	6306
24	5.74	265.1434	[M + H]+	0.0356	C15H20O4	Tabulin	7.8113	101297649
2	12.21	493.1334	[M + H]+	0.6527	C23H24O12	Burkinabin A/C	7.7653	23250812/23250814
25	13.78	533.2748	[M + H]+	−0.2907	C29H40O9	Calactin	7.5948	441849
26	7.67	407.2429	[M + H]+	−0.0847	C23H34O6	Afrogenin	7.5354	N/A
27	14.94	535.2902	[M + H]+	−0.0406	C29H42O9	Gofrusid	7.6154	33647
28	10.15	407.2425	[M + H]+	0.3153	C23H34O6	Palmadorin I	7.5107	71475043
1	10.85	493.1343	[M + H]+	−0.2473	C23H24O12	Burkinabin B	7.8588	23250813

For all the metabolites exact mass, retention time found on the chromatogram and the scoring value obtained using the software are shown. Mass Error was also calculated for each compound using the finding formula on MS-FINDER. Identified peaks present in our in-house plant family databases are represented in blue, whereas identified peaks present in homemade plant genera databases are represented in green.

## Results and discussion

### Optimization of the assay

**Figure 2.**
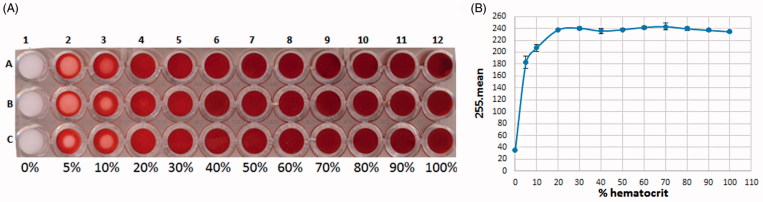
Scanned image of the bottom of a 96-well plate observing color variations with different hematocrit percentages of normal RBC, ranging from 0% (RMPI only) to 100% (A). Corresponding graph representing the variation in mean values subtracted from 255 as a function of the percentage of hematocrit (B).

To obtain a suitable color at the bottom of the 96-well plate, different hematocrit percentages were tested (Figure 2(A)). The corresponding graph representing the mean intensities subtracted from 255 as a function of the percentage of hematocrit is presented in Figure 2(B). Results show that the mean values reached a plateau above 20% hematocrit, indicating that the bottom of the wells was saturated with RBC. On the contrary, in those wells with under 20% hematocrit a lack of RBC caused appearance of white dots. Note that above 50% hematocrit the viscosity of RBC caused pipetting difficulties. Therefore, 30% was selected as the optimal working hematocrit.

The reproducibility of the assay at 30% hematocrit was verified by repeating the experiment for each column of the 96-well plate. Our results showed that the columns 1, 2, 3, 10, 11 and 12 present different values due to a centrifugal effect depending on the position of the well on the 96-well plate. For this reason, these columns were deleted from the working area. An analysis of variance (ANOVA) was performed on columns 4, 5, 6, 7, 8 and 9 (*p* value = 0.42) and demonstrated that the values obtained could not be considered as significantly different. Row A and H were also deleted from the working area to focus experiments in the middle of the plate, so to limit vertical centrifugal effects. On this working area, the calculated *Z*’ Factor was 0.92, indicating that the assay is fit for purpose.

### Kinetics of sickling

**Figure 3.**
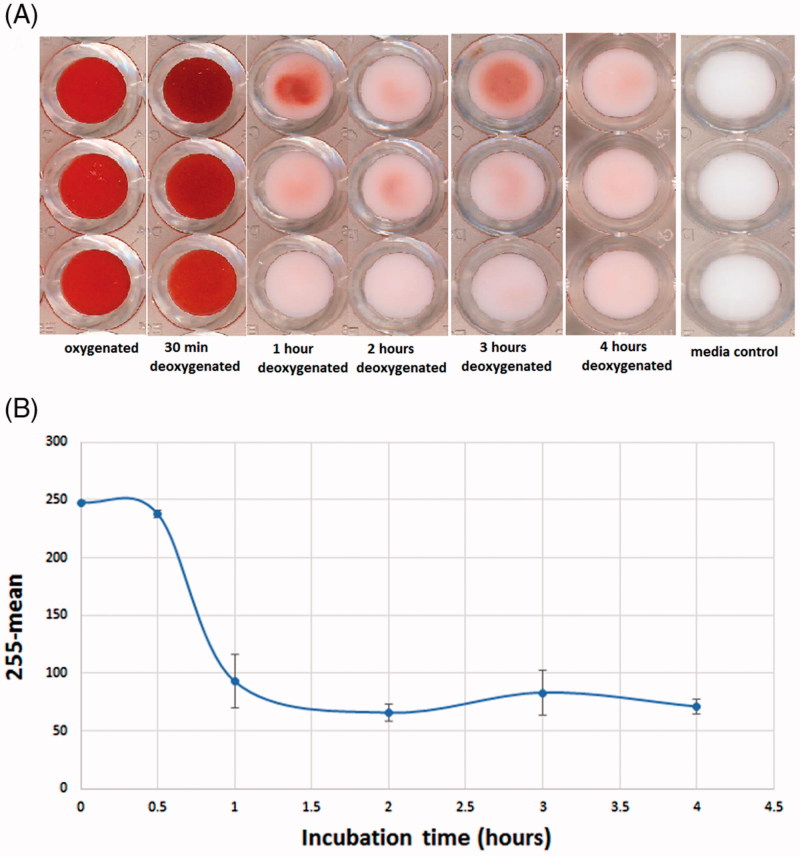
Scanned image of the bottom of a 96-well plate obtained after Hb S RBC were incubated for different amounts of time with sodium metabisulfite (2%) (A). Variation in the rate of differentiation of sickled RBC as a function of incubation time, evaluated as mean values subtracted from 255 (B).

Sickled RBCs were obtained by treating Hb S RBC with sodium metabisulfite (2%). To optimize the incubation time necessary to induce significant transformation of RBC, we performed a kinetic study measuring sickling at different exposure times to hypoxic conditions using the microbead test. We prepared 200 µL of Hb S RBC at 30% hematocrit in PBS (2% sodium metabisulfite) and incubated at 37 °C for 0.5, 1, 2, 3 and 4 h. As demonstrated in Figure 3(A), the induction of sickling was fast: after 1 h of incubation, the bottom of the wells was slightly pink indicating that most of the RBCs in the sample had become sickled RBC. This pink color is due to a small percentage of normal-shaped RBC which did not complete the sickling process. Our results show that 30 min of exposure to sodium metabisulfite is not sufficient to induce differentiation into sickled RBC. As shown in Figure 3(B) a longer incubation time induced additional differentiation, which reaches a plateau after 1 h. For all further experiments, 2 h of exposure to sodium metabisulfite was selected to obtain sickled RBC. Student’s *t*-test was performed between oxygenated control RBC and deoxygenated RBC after 2 h incubation. The *p*-value obtained was 1.21 × 10^−3^, indicating that these two values could be considered as statistically different.

Even if initial results obtained using the microbead assay are encouraging, we observed significant variability, probably dependent on the patients and on the type of treatment that they have previously received for sickle cell anemia. For example, the assay does not work with patients who have just received a blood transfusion; these patients were excluded from the experiment. Treatment with hydroxyurea, which induces the production of Hb F, could also cause some variations in the microbead assay. Hydroxyurea is able to change the percentage of Hb S in RBC, leading to difficulties in obtaining sickled RBC with sodium metabisulfite. This bias could be moderated by using RBC from the same patient for each test. Moreover, deoxygenation of RBC modifies the color of the sample, giving a mauve color instead of a red color. To verify the reversibility of the action of sodium metabisulfite, a positive control was used by diluting normal RBC in contact with sodium metabisulfite in PBS, RBC were able to pass through the microbeads but the red color was not totally recovered, which could result in another bias when the data are processed using ImageJ^®^.

To generate hypoxic conditions, we first tried to use GENbag and GENbag Anaer (Biomerieux, France) (Pascual et al. 2011) in order to mimic physiopathological conditions. But this method was abandoned, due to a lack of reproducibility in obtaining sickled RBC.

One of the advantages of our study is the use of bioinformatics tools which allows us to automate the calculation of means and standard deviation values. This avoids counting sickled RBC one by one on blood smears, which could introduce errors of judgement due to the presence of partially deformed RBC.

### Hemolytic assay of the ethanol aqueous extracts of FACA^®^ and DEPRANOSTAT^®^

Many compounds found in plants could present a risk of hemolysis, for example saponins, which can have a strong hemolytic activity. We evaluated the hemolysis of RBC by measuring absorbance at 450 nm after contact with hydroethanol extracts of FACA^®^ and DREPANOSTAT^®^. Red blood cells in PBS were considered as 0% hemolysis (negative control), whereas RBC in distilled water were considered as 100% hemolysis (positive control). The potential hemolytic activities of FACA^®^ and DREPANOSTAT^®^ aqueous/ethanol extracts were only screened at the high concentration of 1 mg/mL. The hemolysis was quantified using the following formula:

$$\%\text{hemolysis}\;=\frac{\text{Absorbance}\;\text{measured}\;\text{with}\;\text{plant}\;\text{extracts}\;-\text{Absorbance}\;\text{negative}\;\text{control}}{\text{Absorbance}\;\text{positive}\;\text{control}-\text{Absorbance}\;neg\text{ative}\;\text{control}}\times 100$$

$$\%\text{hemolysis}-\text{FACA}=\frac{0.042\;-0.044}{0.947-0.044}\;\times \;100=0\%$$

$$\%\text{hemolysis}-\text{DREPANOSTAT}=\frac{0.088\;-0.044}{0.947-0.044}\;\times \;100=4.87\%$$

Our results showed that the hemolytic activity was low for these two extracts, 0 and 4.87% for FACA^®^ and DREPANOSTAT^®^, respectively. Therefore, these two extracts could be applied to RBC without introducing a bias in the microbead assay, i.e., by changing the quantity of RBC at the bottom of the well for the same hematocrit. The hemolysis occurring upon exposure to DREPANOSTAT^®^ (4.87%) can be considered as negligible.

### Effect of plant extracts on sickled red blood cells

To evaluate the anti-sickling potential of the two CAM, we incubated Hb S RBC (30% hematocrit) for 2 h with sodium metabisulfite and screening concentrations of hydroethanol extracts of FACA^®^ and DREPANOSTAT^®^ from 0.2 to 1 mg/mL. Plant powders were dissolved using PBS containing 2% sodium metabisulfite in order to maintain the percentage of this agent in solution. The percentage of sickling was then calculated by comparing oxygenated Hb S RBC without sodium metabisulfite (positive control) and deoxygenated RBC without plant extracts (negative control) using the following formula:

$$\%\text{sickled}\;RBC=\frac{\text{Mean}\;\text{positive}\;\text{control}\;-\;\text{Mean}\;of\;the\;\text{screening}\;\text{concentration}}{\text{Mean}\;\text{positive}\;\text{control}-\text{Mean}\;\text{negative}\;\text{control}}\;\times \;100$$

**Figure 4.**
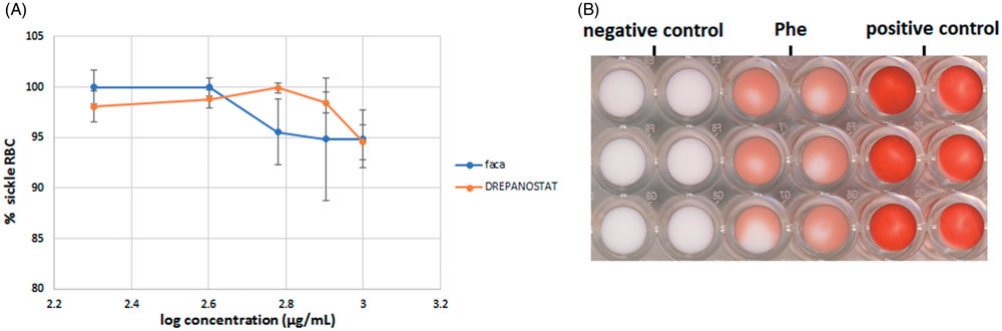
Variation of the percentage of sickled RBC as a function of the concentration of extracts of FACA^®^ and DREPANOSTAT^®^ (A). Scanned image of the bottom of a 96-well-plate obtained with sickled RBC previously incubated with 7 mg/mL of phenylalanine (Phe) (B).

Results presented in Figure 4(A) clearly show that even at 1 mg/ml, the ethanol aqueous extracts of FACA^®^ and DREPANOSTAT^®^ have very weak anti-sickling effects. Even at this concentration, more than 90% of the total RBC were blocked at the top of the microbeads, indicating that most of them are sickled RBC. When comparing the plant extracts to the negative control, the *p*-values were 0.64 for Hb S RBC treated with 1 mg/mL FACA^®^ and 0.077 for 1 mg/mL DREPANOSTAT^®^. Therefore, no significant difference was found between the mean values obtained with the plant extracts and the negative control, even at this high concentration. Thus, the extracts did not inhibit RBC sickling in these conditions.

Moreover, as seen in Figure 4(B), we used a high concentration of phenylalanine (7 mg/mL) as a control in this assay, as its anti-sickling effect has previously been described (Pais et al. 2009). We demonstrated that the percentage of sickled RBC was reduced to 52% after treatment with phenylalanine; therefore our microbead assay could easily identify new anti-sickling agents. However, our assay did not allow us to demonstrate the activity of FACA^®^ and DREPANOSTAT^®^, since no significant inhibition of RBC sickling was obtained after incubation in the presence of the hydroethanol extracts. This low anti-sickling effect could be due to too large a number of molecules being screened, resulting in a matrix effect i.e., a large number of screened molecules in the extract masking the potential anti-sickling effect of a molecule. A fractionation of these two extracts is in progress in order to reduce the number of molecules in contact with RBC.

We also performed an experiment to measure the reformation of sickled RBC after 24 h incubation with the extracts (0.2–1 mg/mL). Unfortunately, we were unable to conclude the experiment due to the aggregation of RBC after 24 h incubation, and a kinetic study is in progress to optimize the incubation time so to avoid aggregation.

A limitation of this test is that it only targets sickled RBC, whereas is has been demonstrated elsewhere that plant extracts may also act on other blood components (endothelial cells, macrophages etc.) to have anti-occlusive effects. For example, published work has highlighted the vasodilatation effect of FACA^®^ (Ouedraogo et al. 2011). To evaluate the action on endothelial cells, a new microfluidic test inspired from a previous study (Mannino et al. 2015) is currently under development. The aim of this test is to use a microfluidic device (microslide Y shaped IBIDI) covered with endothelial cells to mimic blood vessel bifurcation (Moonen et al. 2015). The rate of rhodamine 18 labelled-RBC blocked in the channel can then be evaluated by measuring the fluorescence. By combining results from the microbead assay used in this study and this new microfluidic test, it would be possible to form hypotheses about the action of tested plants on RBC and on endothelial cells.

### *In silico* analysis of extracts

Bio-guided fractionation is a conservative method used to isolate potentially active components, however it is time-consuming. Substantial work has already been carried out on many different families and genera of plants, subsequently relatively complete databases are now available. Thanks to the development of bioinformatics, it is now possible to search for potential active metabolites using databases containing compounds previously isolated. This is a faster approach with relatively low uncertainty.

As seen in Table 1, we identified 28 metabolites in FACA^®^ and DREPANOSTAT^®^ that had scoring values above 7.5 and were in agreement between the screened databases. In total, 520 integration peaks were realized for DREPANOSTAT^®^ in the positive and negative ionization modes. Among them, 270 were identified in MS-FINDER using the plant databases, but only 64 of them had a scoring value above 7.5. Finally, only 19 of them were in agreement with our in-house databases. For FACA^®^, 467 integration peaks were realized with MS-DIAL in the positive and negative ionization modes. Among them, 284 were identified in the plant databases and 55 of them were retained with a scoring value above 7.5. Only 16 of them agreed with our in-house databases. This low number of identified metabolites is due to our strict filtering method, selecting only those metabolites with high scoring values and that were in agreement between databases. As a result, the identification of false positives was strongly reduced.

A few of the metabolites presented in Table 1 have previously been isolated and identified (Carboni 2009; Chaaib Kouri 2004), from the same family of plants but sometimes from different genera. The most interesting identification in this list are burkinabins A/B/C (ID: 1; 2) which have been identified as anti-sickling agents from the root bark of *Z. zanthoxyloides* (Ouattara et al. 2004, 2009). A protocol has been described in the literature to separate these three molecules, however the yield from this extraction is very low and the raw material required (DREPANOSTAT^®^; which is composed only of *Z. zanthoxyloides*) is too important to experiment with. Some amino acids have also previously been identified as anti-sickling agents, such as tryptophan, isoleucine and phenylalanine (ID: 12; 23; 8), and it has been shown that at high concentrations phenylalanine is able to reverse sickling (Pais et al. 2009); a finding which has been corroborated in this study.

The identification of calactin (ID: 25) is particularly surprising because of its previously described anti-proliferative effects (Lee et al. 2012). Chronic administration of FACA^®^ is used for the prevention of pain crisis, however long term exposure to calactin could cause side effects for patients.

In the future, we aim to apply fractionation methods to identified active vegetal extracts. Indeed, all fractions could be tested using the microbead assay to obtain an activity profile. Finally, LC-MS/MS would allow us to link the most active fraction to the corresponding peaks at a specific retention time. This also limits the number of bioactive candidates and reduces the total number of metabolites potentially interacting with RBC, which could improve the effect of the bioactive molecules on RBC. Indeed, the low anti-sickling effect we observed with extracts of DREPANOSTAT^®^ and FACA^®^ could be explained by a too high number of molecules being screened, resulting in a matrix effect.

## Conclusions

The assay we developed in this study presents several advantages since it is fast, inexpensive and requires only a small quantity of raw material, and therefore could be easily transposed to sub-Saharan laboratories. Even though the experiments we carried out in this work did not allow us to demonstrate the anti-sickling activity of FACA^®^ and DREPANOSTAT^®^, the assay we developed can easily measure the inhibition of RBC sickling. This difficulty could be due to the complexity of the chemical extract differing from the drug metabolites present in patients. Hence, it would be interesting to isolate the compounds identified as being the most active. Our assay has only been tested on the extracts of two African plants, and locally they can be scarce. However, other African plants have also reported to have anti-sickling properties (Ameh et al. 2012; Mpiana et al. 2007). Our assay could be used on extracts of these plants to verify their anti-sickling effects, and attempts could be made to try and identify any bioactive molecules. The eventual anti-sickling effects of synthetic molecules could also be evaluated using our assay.
